# The African Gender and Development Index: an engendered and culturally sensitive statistical tool

**DOI:** 10.3389/fsoc.2023.1114095

**Published:** 2023-06-01

**Authors:** Jacques Charmes, Saskia E. Wieringa, Thokozile Ruzvidzo, Gonzaque Rosalie

**Affiliations:** ^1^Institute of Research for Development (IRD), University of Paris, Paris, France; ^2^Amsterdam Institute for Social Science Research (AISSR), University of Amsterdam, Amsterdam, Netherlands; ^3^Consultant, Addis Ababa, Ethiopia; ^4^Economist at UNECA, Addis Ababa, Ethiopia

**Keywords:** gender statistics, Africa, SGDs, GDI, GEM, index

## Abstract

In 2004 the African Union adopted an innovative gender index, the Afriacn Gender and Development Index (AGDI). It is composed of the quantitative Gender Status Index (GSI) and the qualitative African Women's Progress Scorecard (AWPS). The tool is built on the use of national data collected by a national team of specialists. Since the beginning three cycles of implementation have occurred. After the last cycle the AGDI was revised. In this article the authors assess the implementation of the AGDI, against the background of other gender indices, and discuss the latest revisions.

## 1. Introduction

Development that is not engendered is endangered was the slogan of the 1995 issue of the Human Development Report of the UNDP (United Nations Development Programme). It was a powerful conclusion, which has lost none of its value since then. The 1995 UNDP report had been drafted in preparation for the Fourth Women's World Conference, held in Beijing, September 1995. It presented two new complementary indices, the Gender-Related Development Index (GDI) and the Gender Empowerment Measure (GEM) (UNDP, 1996). Since this path breaking report gender indices have become increasingly influential tools for governance and knowledge production. The first indices of the UNDP tried to break through the dependence on the Gross Domestic Product (GDP), characteristic of earlier international development indices and to measure capabilities and opportunities of human beings, following the work of Amartya Sen.^1^ In 1990, inspired by Sen's work, the UNDP had already introduced the Human Development Index (HDI), a composite index in which human development is ranked. It is based on three dimensions, a long and healthy life, being knowledgeable and having a decent standard of living. The HDI was the arithmetic mean of normalized indices for each of these three dimensions.^2^ This index was the starting point of a never-ending popularity for composite indices.

The World Bank agrees that gender inequalities impede development. A 2001 report concludes that “ignoring gender disparities comes at great cost to people's wellbeing and to countries' abilities to grow sustainably, to govern effectively, and thus to reduce poverty. This conclusion presents an important challenge to the development community” (Mason and King, 2001).

Regular reporting requirements to international bodies that measure progress on gender equality also make it imperative to produce internationally comparable engendered data. These reports include the regular cycle of reporting (every 4 years for individual countries) on the CEDAW (Convention on the Elimination of All Forms of Discrimination against Women) which was adopted by the UN General Assembly in December 1979. A growing list of countries has since ratified this Convention, many with the additional Optional Protocol.^3^ Following the Millennium Development Goals (MDGs), the Sustainable Development Goals (SDGs) were adopted by the UN in 2015. They entail a call to end poverty, to protect the planet and to ensure that by 2030 all people enjoy peace and security. SDG 5 calls to achieve gender equality and empower all women and girls. Various other SDGs also include gender components.^4^ Eliminating all forms of violence against all women and girls in the public and private spheres is an important component of SDG 5.

The GDI is the HDI disaggregated for gender. This first effort at engendering global statistics inspired researchers and statisticians all over the world to assess the GDI and the GEM and to design new gender indices. In this article we present some of the most relevant points of critique on the GDI and the GEM. We also give a short overview of some of the major gender indices that were produced in the following decades in order to highlight the innovative design and the inspiring implementation of the African Gender and Development Index (AGDI), a process in which the authors were deeply involved. We discuss the Global Gender Gap Index (GGDI) launched by the World Economic Forum (WEF), the OECD's Social Institutions and Gender Index (SIGI) and the Gender Equality Index (GEI) of the EU. We also mention the new gender index of the UNDP, the Gender Inequality Index (GII), the Power of Parity Index, and the UNDP's Gender Social Norms Index and the SDG Gender Index.

The focus of this article is on the design and implementation of the AGDI. While the compilation and computation of composite indices are exercises that generally intend to cover as many countries as possible across regions and sub-regions, increasing the risk of non-pertinence, the experiences of indices focussing on a particular region have been scarce. The AGDI conceived by the Gender and Development Center of the UN Economic Commission for Africa (UNECA), was the first regional index, developed from the early 2000s. The only other regional index is the European GEI, developed from 2005.

### 1.1. Discussions on the GDI and the GEM

At the request of the Dutch Ministry of Foreign Affairs in 1996–1997 a project was launched by the Institute of Social Studies in The Hague, to assess the value of the GDI and the GEM for three developing countries of roughly the same size as The Netherlands, namely Benin, Bhutan and Costa Rica.^5^ A major conclusion was that though these countries might be considered more advanced in particular aspects of gender equality, The Netherlands came out on top, due to the overriding weight of the income variable. The GDP remained dominant. Also, it was felt that the selected indicators were limited in scope (Wieringa, 1997).^6^ In further efforts of the same team alternatives such as a Women's Empowerment Matrix (WEM) and indicators for a Gender Equality Index (GEI) were proposed (Wieringa, 1997; Dijkstra and Hanmer, 2000; Dijkstra, 2002).^7^

Later critics agreed that the GDI and the GEM measured general socio-economic welfare based on national income rather than gender equality. The GDI measures the overall development levels in a given country corrected by the existing gender inequalities. The GEM measures the extent to which women have access to certain levels of power, in which the absolute income level per capita still weighs heavily (Dijkstra, 2002).^8^ Other points of critique on the GDI and the GEM include the limited conceptualization of gender power and women's empowerment (Wieringa, 1998, 2006).^9^ Many issues relevant for women's empowerment, such as compliance with CEDAW as well as other human rights, cultural and legal forms of women's subordination and issues related to the body and sexuality were left out of the GDI and the GEM. A further issue was the use of international rather than national databases such as those of the ILO or UNESCO. These data were outdated by the time they were presented, as these international organizations first had to collect the national data and harmonize them for international comparative use. In the process much national specificity got lost due to the complicated harmonization processes required to compare various national data sets. Further it was noted that several indicators had flaws; longevity for instance, the indicator used to measure health, is a stock indicator and slow to change. For income only non-agricultural wages in the formal sector counted, ignoring informal labor, care work and the agricultural sector. The indicator for political power (share of women in parliament) was also seen as insufficiently measuring women's power or the lack of it (Charmes and Wieringa, 2003).^10^ Women-specific indicators could not be collected, such as the Maternal Mortality Rate (MMR). Last, the deployment of the complex statistical calculations used in the GDI and the GEM puts the analysis of the indices practically in the hands of statistical bureaus, limiting their relevance for the civil society sector.

## 2. Indices since the GDI and the GEM

Since the GDI and the GEM have been introduced, several other organizations have introduced gender indices; more than 15 different gender indices have been designed.^11^ These include the Gender Equality Index of the European Union, published in 2013 and the Global Gender Gap Index computed for the first time in 2006 by the World Economic Forum. In 2010 the UNDP itself has also introduced a new gender index, the GII. In 2012 the OECD launched the Social Institutions and Gender Index (SIGI). The one-shot McKinsey Global Institute Gender Parity Score (GPS) was released in 2015. In 2019 the UNDP designed a Gender Social Norms Index (GSNI) to accompany the GII and “Equal Measures 2030”—a think tank and advocacy network—presented the SDG Gender Index (SDGGI) in 2022 (Equal Measures 2030, 2022). In 2010 the World Bank launched its flagship report “Women, Business and the Law.” In this article we discuss them only in relation to their relevance to the AGDI. We first discuss the advantages and disadvantages of indices and of ranking and the deployment of international databases and of microdata sets. We conclude the section with a brief discussion on the various gender indices in Africa, introduced after the AGDI (UNECA), namely the Africa Gender Index (AGI) of the African Development Bank (AfDB) and the United Nations Economic Commission for Africa (ECA) and the Africa Gender Scorecard (AGS) of the African Union Commission (AUC). The focus will be on issues of competition, complementarity and cooperation.

This popularity of composite indices and their extension to all countries of the world, notwithstanding regional and country specificities, raise several issues that are pros and cons for the compilation and use of composite indices: namely the availability of comparable data, the issue of possible conflicts between national data and international databases, the meaning of concepts and indicators, the issue of ranking and the risk of intervention to influence the ranking.

### 2.1. Issues in compilation and use of composite indices

#### 2.1.1. Availability of comparable data

Mainly due to lack of resources, certain regions and certain countries are not collecting—or at least are not collecting on a regular basis—some of the common indicators that are widely available across regions. This is the case for employment: the number of African countries that conduct labor force surveys on a quarterly or even a yearly basis remains low. Generally, the living conditions surveys provide such indicators on an irregular or 5-year basis. In recent years, some efforts of harmonization have been made, for instance the harmonized household living conditions survey in Western Africa. Sometimes a specific concept is not applied in certain regions: an example is the concept of informal employment—highly significant for defining labor markets in developing countries—that does not give rise to data collection in developed countries and must therefore be replaced by a substitute or a proxy. Or while civil registries and health statistics provide detailed information in developed countries (but do not capture female genital mutilation), the Demographic and Health surveys (DHS) collect demographic and health status data in Africa, including on female genital mutilation and other details on reproductive health that remain unknown in developed countries. For these reasons databases are not strictly comparable from country to country and from year to year. Hence the usefulness of international databases.

#### 2.1.2. Possible conflicts between national data and international databases

International institutions have early built databases for the indicators they are responsible for defining, classifying and harmonizing, for instance the International Labor Organization (ILO) for labor force and employment (ILOSTAT),^12^ the UNESCO Institute of Statistics for education statistics,^13^ the World Health Organization (WHO) for health statistics (Global Health Observatory).^14^ The World Development Indicators of the World Bank^15^ gather all (or most) of the indicators collected by the other international institutions complemented with its own sources. And since the adoption of the SDGs in 2015 the UN database for SDGs^16^ gathers all the 231 unique indicators defining the goals; these are supplied and maintained by other international institutions. More recently, some of these databases have gathered the microdata of the related surveys and have now the possibility of generating the indicators along various definitions and algorithms that may diverge from those used at national level.

All these improvements have meant that reliance on and references to national data may seem redundant or unnecessary. This is a mistake. Although international institutions are in charge of definitions of concepts through international conferences that adopt resolutions, recommendations, guidelines and methodologies, the reality of data collection and metadata in the field makes that national practices and specificities do not always reach international databases and that misunderstandings and misinterpretations are always possible. Not to mention the risk of preferring less reliable but more accessible surveys, or that some countries may not officially recognize a concept or that national definitions may not coincide with international definitions for some reasons that should not be underestimated or neglected. For instance, the indicator for informal employment included in the SDGs database was overestimated by more than 20 percentage points in an African country due to the misinterpretation—at international level—of one question in the labor force survey.^17^

#### 2.1.3. Issue of ranking and risk of intervention to influence the ranking

The yearly ranking of the Human Development Index has often given rise to nervous breakdowns, headaches, or fits of anger on the part of national policy-makers and politicians, anxious to see the ranking of their country as compared with neighboring countries or the deterioration in ranking whereas they were convinced to have taken the right decisions to improve the ranking. Interventions to influence the ranking may also occur, as exemplified by what happened to the World Bank flagship report “Doing Business.” This has led to the interruption of this publication, which was influential in that levels and trends in countries' rankings reported therein were constantly referred to in IMF and World Bank reports.

Although the above reflections may seem far from the object of this article, we will see that the approach adopted for conceiving and implementing the AGDI was well aware of the inherent risks long before they became obvious.

### 2.2. Variety of composite indices since the GDI and the GEM

#### 2.2.1. Gender Equality Index

Since 2005 the European Union has developed its Gender Equality Index (GEI). It is intended as a tool to measure gender equality in ways that reflect the specific conditions of the EU needed to stimulate effective policymaking on gender issues. The first edition appeared in 2013. Besides reflecting socio-political sensitivity, the index was meant to measure the complexity of gender equality in a user-friendly way. It is divided into six domains (work, money, knowledge, time, power and health), which are again divided into subdomains which each have several indicators. The overall score is calculated as a combination of arithmetic (at variable level), geometric (at sub-domain level) and harmonic (at domain level) means.^18^ Two satellite domains (intersecting inequalities and violence) which are not included in the calculations of the index provide extra information. Interestingly the index has various indicators that are particularly relevant for countries in the European Union, such as the gender gap in pensions and in unmet needs for dental examination.

The first report of the GEI noted that in the EU the greatest challenges to gender equality were the unequal division of time for childcare, domestic and leisure activities between women and men (this form of inequality increased since 2005) as well as the representation of women in power and decision-making positions.^19^ In 2015 the GEI report compared progress between 2005, 2010, and 2012.^20^ This report indicates a slight improvement in various dimensions of gender equality. The 2013 report identified the domain of violence as the greatest gap in data collection because of a lack of comparable and harmonized data at EU level. It is indeed difficult to calculate this indicator, as levels of reported gender-based violence may not indicate incidence of violence. Higher levels of reported violence may point to the success of feminist movements or the increased sensitivity of police and justice institutions.^21^

However, as Permanyer (2015) observed, the index measures not so much equality, but rather the relative achievement of women in relation to men in the various domains. He concludes that the GEI scores largely reflect differences in overall achievement levels *between* countries rather than gender differences *within* them. This is a similar critique as that directed at the GDI, which measures overall wellbeing based on levels of GDP rather than the gender gap within and between countries. Thus, in the GEI low-income countries are penalized for factors that are not related to gender regimes. This compromises the utility of the GEI for designing gender policies. The choice of indicators for the domain of power, with its focus on top level positions in various socio-economic realms is also noteworthy. Political power and power in the judiciary are ignored.^22^

#### 2.2.2. World Economic Forum: Global Gender Gap Index

The aim of the World Economic Forum (WEF) to develop the GGGI was to avoid the reproduction of a gap between poor and rich countries. The first report was produced in 2006; the GGGI has been published on a yearly basis since then. The GGGI defines gender equality as equal rights, responsibilities and opportunities.^23^ Compared to the GDI and the GEM this first report offered a more detailed measurement of gender equality, combining 14 indicators across health and survival, educational attainment, political empowerment, and economic participation and opportunities. The GGGI is a one-sided scale, gender imbalances in which women score better than men do not affect the calculation. The only exception is the (flow) indicator of longevity, in which it is assumed parity is reached when women live 5 years longer than men. The GGGI is heavily dependent on international data bases, such as those of the ILO, UNESCO, WHO, and the OECD, similar to the UNDP's gender indices.

The focus on gender equality and gaps in access to resources and opportunities within countries avoids to a certain extent the dependence on national income. But it also fails to measure women's empowerment. Problematic is the limited conceptualization of power. Only the ratio of women to men in minister-level positions, the ratio of women to men in terms of years in executive office (prime minister or president) in the last 50 years and the ratio of women to men in parliamentary positions are examined. This leaves out vast areas of power, particularly in the judiciary, and at subnational levels.^24^ An interesting indicator is the sex ratio at birth. This captures the phenomenon of “missing women,” characteristic of many countries with strong son preference. In the 2021 report the WEF lists other interesting variables, such as the rights of women to travel outside the country, to equal justice, to divorce and to inheritance.^25^ The MMR and intimate partner violence are also included.^26^ For income the rates for formal employment are used. In the absence of such data a proxy of 0.75% is applied.^27^

#### 2.2.3. UNDP Gender Inequality Index

In 2010 the Human Development Report (UNDP, 2010) replaced the GDI and GEM with the Gender-adjusted Human Development Index and the Gender Inequality Index (GII). The GII is a composite metric of gender inequality using three dimensions: reproductive health, empowerment and the labor market. A low GII value indicates low inequality between women and men, and vice-versa.^28^ The computation is very complex; it entails seven steps each with different formulae, leaving the index under the control of statistical offices. Though the use of only five indicators is attractive in its apparent simplicity, it also means that a lot of relevant information is left out. The share of women in parliament for instance is a poor indicator of women's empowerment, leaving out the judiciary and power at the local level. Though the use of the labor force participation rate can be seen as more reliable than data on wage gender gaps, it underestimates informal employment and unpaid care work. Critical issues such as access to credit and land, gender-based violence and compliance with laws and regulations are ignored. Permanyer (2013) concludes that the “functional form of the index is excessively and unnecessarily confusing. Moreover, the inclusion of indicators that compare the relative performance of women vis-à-vis men, together with absolute women-specific indicators, obscures even more the interpretation of an already complicated index and penalizes the performance of low-income countries.” These limitations “limit the usefulness and appropriateness of the GII as a global gender index.”

An analysis of the exercise of the Indonesian National Statistical Bureau to produce a subnational GII for all provinces revealed several weaknesses of this new UNDP gender tool (Wieringa, 2019). First of all, the GII is not transparent. Its single figure outcome obscures underlying problems with the selection of indicators and data collection. An example is the computation of the MMR. As it was found that data on the very high MMR of Indonesia are not reliable, the GII uses a proxy, the proportion of women who don't give birth in medical facilities. This is insufficient. Indonesia's high MMR is not only related to the availability and use of trained midwives and health facilities, but also to structural inequalities in the gender-based division of labor, leading to overwork, and anemia.^29^ For political power only the share of women lawmakers is measured, while power at the lower levels, up to that of the village as well as in the judiciary is ignored. Lastly the gender gaps in wages and income, and in education level from the junior high school upwards are ignored.

#### 2.2.4. Social Institutions and Gender Index

The Organization for Economic Co-operation and Development (OECD) uses a different approach to assess gender-based inequalities. The Social Institutions and Gender Index (SIGI) was designed by the OECD Development Center in 2009, with three updates in 2012, 2014, and 2019 (OECD Organisation for Economic Co-operation and Development, 2014; OECD, 2019). It measures the level of discrimination in laws, social norms and practices and comprises four dimensions, 16 indicators and 27 variables. The SIGI was initially conceived for describing women's status in developing countries (non-OECD and non-EU countries) and some of its indicators are less pertinent and not measured in developed countries, except for migrant populations from developing countries (child marriage, female genital mutilation for instance).

The SIGI is a composite index that provides a cross-country measure of discriminatory social institutions, which includes formal and informal laws, social norms and practices that restrict women's and girls' rights, as well as their access to empowerment opportunities and resources. Rather than measuring outcomes, it focuses on the institutions that underlie discriminatory practices. The SIGI is based on analyses of the following dimensions: the family code, civil liberties, physical integrity, and son preference and ownership rights. The impact of long-lasting social institutions on societal practices and legal norms is measured to assess the extent of women's subordination. It is a very useful tool that makes available additional information to that supplied by the indices discussed above.^30^

The 2021 regional report for Africa provides data for the 54 African countries (OECD, 2021). It exposes discriminatory laws, norms and practices. Progress is noted in women's political leadership and participation in decision-making, particularly in countries that implement affirmative practices such as quotas. But high levels of discriminatory practices persist, based on male dominance in the private sphere and women's acceptance of that. Child marriages, female genital mutilation (FGM) and intimate partner violence continue. The distribution of unpaid care work is unequal, while women's land rights are restricted. The OECD index is useful by focusing on the origins of the inequalities that have become manifest in indices which are outcome-based.

There are several problems with the SIGI. In the first place the information on sexual violence is based on laws only, as data on prevalence were not available. But there may be a big gap between a good law and its successful implementation. The same goes for access to land, credit and property other than land. Only legal rights are considered, not the actual prevalence. The SIGI does not measure implementation. There are other omissions. In the dimension for physical integrity abortion is not considered. There is also no indicator on political institutions, so that gendered power inequalities remain under the radar.

Innovative as the SIGI is, quantifying qualitative issues is a big challenge. Country teams themselves are not involved in the coding of the sub-indices, this is done centrally in the OECD Development Center. The complexities of the formulas impede the direct deployment of the SIGI by local NGOs. It is difficult to assess the impact of data limitations, the choice of particular indicators, or their proxies and dimensions. Son preference, female genital mutilation and dress codes for women are relevant in particular countries, but not in others. Again, good laws may not correspond to actual prevalence of these customs. Also, heteronormative biases in the choice of indicators related to the assessment of the family codes may lead to countries that are extremely homophobic (such as Jamaica and Belarus) scoring very high on this dimension.

#### 2.2.5. Other recent global indices

The Power of Parity Index or Gender Parity Score (GPS) of the McKinsey Global Institute (2015) ends up with an estimate of 12 trillion US$ that could be added to GDP if gender equality were achieved. The index is comprised of 15 outcome-based indicators (some of them being composite indicators, so that at the end, 28 indicators are mobilized) gathered for 95 countries. These indicators are distributed through several blocks and scrutinized around several global and regional “impact zones”: “gender equality in work” and “gender equality in society,” subdivided in “essential services and enablers of economic opportunity,” “legal protection and political voice” and “physical security and autonomy.” Using female-to-male ratios and including unpaid care work, this index is typically following the same approach as the GSI of AGDI with a different distribution and aggregation of similar indicators within blocks, but rather than ending with a precise figure of the index and sub-indices, it provides the results along four levels of gender inequality: extremely high, high, medium and low, and it regroups countries by region along these four levels. Individual countries are never ranked but rather positioned on two-ax graphs. The GPS is not intended to be computed regularly and is rather a one-shot exercise.

In the 2019 Human Development Report, the UNDP introduced the Gender Social Norms Index (GSNI). The index (UNDP, 2020) is built around four dimensions: political, educational, economic and physical integrity that are informed through the responses to questions from the World Values Survey. For example: “Men should have more rights to a job than women.” It is therefore a qualitative index based on opinions about gender stereotypes.^31^ The quantification of indicators is operated through several choices: agree/disagree, essential/not essential and along a scale from 1–2 to 1–10. Results are displayed in percentages of women and men with at least one bias, at least two biases and no bias.

Lastly, the broadest cross-cutting database gave rise to the compilation of a new gender index: the SDG Gender Index (SDGGI) was launched in 2019 by Equal Measures 2030. Its second edition in 2022 covers 58 indicators across 14 of the 17 SDGs for 144 countries (135 with two time points) and more in depth for seven focus countries (Burkina Faso, Colombia, Guatemala, India, Indonesia, Kenya and Senegal) and 3 focus regions (Africa, Latin America and Asia). It is unique in that it follows the structure of the SDGs with a gender lens, thus tracking progress across most of the goals; it also complements the gaps by drawing on a wider range of data sources including legal frameworks. However, one can note that time spent in unpaid care work was dropped among the indicators for SDG 5. Countries are ranked by their scores, but also distributed into five categories for levels (from very good to very poor) and 4 categories for progress (from fast progress to decline). According to the SDGGI, the final global score reached 68 in 2020 and could reach 71 in 2030 as compared to 100 for the 2030 targets. The gendered effects of Covid, the Russian war against Ukraine and climate change may further imperil the goal of reaching the SDGs' target.

### 2.3. Gender indices in Africa: competition, complementarity and cooperation: from AGDI (UNECA) to AGI (AfDB) and AGS (AUC)

In 2015–2016, while AGDI was entering into its round of covering 39 countries, the African Development Bank launched the African Gender Equality Index (African Development Bank AfDB, 2015), repeated (and widely changed) in 2019 (AfDB and UNECA, 2020) and currently under repetition (in 2022). The African Union Commission presented the African Gender Scorecard (AGS) (African Union Commission AUC, 2015), repeated in 2016 (AUC, 2017), and in 2021. These initiatives show that despite the efforts of the AGDI programmes, the need for an overall picture of the continent could not prevent some kind of duplication. For the repetition of their respective index and scorecard, the AfDB and the AUC associated themselves with the UNECA in order to avoid competition and duplication, to benefit of the lessons learned from the AGDI experience and to reach some forms of cooperation that could lead to a simplified and unified tool at continent level.

The AGI of the AfDB and UNECA (2020) retains the ratio of female to male achievement in the three dimensions of the quantitative GSI of AGDI, defined along Sen's theoretical and analytical framework: economic (opportunities), social (capabilities) and representation and empowerment (agency). Within these three dimensions, sub-components are defined (three for economic, with eight indicators; two for social, with six indicators; and four for representation and empowerment, with five indicators). Indicators were selected on four main principles: soundness, measurability, country coverage and relevance to the phenomenon being measured and relationship to each other. In 2019 the AGI scores range from 24 to 79.7% with an average of 48.6%, yielding an overall gender gap of 51.4%.

The AGS of AUC (2017) also retains the ratio of female to male achievement and focuses on three clusters of women's rights: economic, social, civil and political. It privileges national data. Its main originality resides in that it distinguishes between three types among the 23 indicators that are used for the three dimensions: input indicators, also called resource indicators (qualitative indicators that refer to the existence of legislation or policies that advance women's rights and their operationalization in practice: for instance maternity leave); output indicators that are quantities produced or numbers achieved (for instance, number of beneficiaries of maternal health services or victims of domestic violence); and outcome indicators that are quantitative indicators measuring the impact or effect of the implementation of legal frameworks in terms of prevalence, participation rates, changes and benefits (for instance access to water and sanitation). Another originality of the AGS was its graphic presentation mapping the indicators for the 51 African countries covered. Moreover, in order to avoid the issue of ranking, the AGS nominated countries above average in each dimension and selected one or two on top for awards, so that several countries received awards for achievement and progress in the various dimensions of the scorecard.

The two indices of the AfDB and the AUC show that they are indebted to the AGDI and while their first rounds (2015 AGI and 2016 AGS) were conducted independently, the UNECA was involved in their second round. However, the lack of annual data makes it difficult to produce a yearly index and leads to focus on specific issues to enrich the index and the list of indicators.

## 3. A culturally sensitive and feminist index: AGDI

The African Gender and Development Index (AGDI) by the United Nations Economic Commission for Africa (UNECA) was conceptualized from 2000 on.^32^ The challenge was to design a gender index with which African countries could be compared among themselves, that would capture the quantitative aspects of gender relations as well as sex specific issues such as the MMR. It should include the relevant quantitative and qualitative dimensions of women's empowerment as well as the implementation of the various international charters and conventions related to gender issues in Africa. The level of national income should not be leading. Apart from these considerations the AGDI was built on a conceptual framework consisting of two pillars. These are the capabilities approach introduced by Sen, and the women's empowerment approach created by gender and development specialists. The capabilities approach deals with potential “functionings,” focusing on freedoms and opportunities that women and men have to lead the lives that they have reason to value—what Sen refers to as “doings and beings.” In assessing human welfare and inequality in economics, this theory goes beyond a reliance on a country's GDP for comparative reasons. A country's development then is measured in expanding the capabilities of its population in terms of a long healthy life, being knowledgeable and having a decent standard of living.^33^ The UNDP in its 1995 report translated that into indicators for health, education and income.

The women's empowerment approach was built on studies developed from the late 1970s on the distinction between sex (biological) and gender (social).^34^ The gender and development school emphatically stresses that economic development alone is not enough to bring about gender equality, though gender inequality may be reduced as a result of economic development.^35^ Scott defined gender as a social relation of power, which operates in a number of realms, from the personal to the cultural.^36^ Women's empowerment as suggested by Kabeer (1999) deals with access to resources, the increase of women's agency and achievements of women in several realms, such as education. These insights are translated in the AGDI in for instance a broader definition of political power, and the incorporation of variables dealing with violence, sexuality and a broader range of economic variables.

Another influential feminist theory was contributed by Haraway (1988). She refuted the objectivity claim of scientific knowledge and posited that all forms of knowledge are situated. This helped justify the building of an African gender index, built on the realities as experienced by African women and men. In building and implementing the AGDI we stressed the positionality of the researchers and African gender experts involved in the process.^37^

Before the AGDI was adopted as the tool to measure the gap in status between women and men and to monitor progress toward women's empowerment, a lengthy process of consultations took place. A working group gave valuable suggestions. A regional advisory panel composed of representatives of the 12 countries where the AGDI would be piloted and representatives from various international bodies reviewed and validated the draft AGDI. In the process of selecting variables, we naturally encountered the usual dilemmas of choosing between available indicators and desired indicators. In several cases we decided to include indicators (for instance on time use) for which we knew not all countries collected data. Pointing to the gaps in the collection of indispensable data might make countries more willing to collect them.

In 2002 a first report on the AGDI appeared (UNECA, 2002). It spelled out the following objectives:

- To provide African policy makers, gender planners and politicians with an appropriate tool to measure the extent of the gender regime in their countries and the effects of their policies to reduce women's subordination;
- To measure the gender gap between women and men irrespective of a country's socio-economic development;
- To make use of nationally available data;
- To monitor the progress made in the ratification and implementation of CEDAW and other conventions and charters related to women's rights;
- To democratize statistics by providing a valid, effective, reliable and easy to use monitoring tool to stimulate the process of community participation and enhance political awareness of gender concerns;
- To provide a tool that measures women's empowerment in qualitative and quantitative terms to capture the complex and dynamic reality of African women's lives.

A composite index was designed, consisting of the quantitative Gender Status Index (GSI) and the qualitative African Women's Progress Scoreboard (AWPS). Data collection should be implemented by national teams, from countries that expressed interest in participating in the AGDI process. The UNECA offered consultancy and training to support data collection and the development of national AGDI reports. In this way national ownership of the AGDI could be guaranteed, while international comparability could be maximized. Those teams should consist of statisticians and gender experts; its methodology should be transparent and as simple as possible. After the index was endorsed by African states in 2004, it was first piloted in 12 countries in 2004–2005. The outcome report was published in 2009 as a special issue of the African Women Report (UNECA, 2009). In a second, third and fourth round the AGDI was computed by 41 countries (UNECA, 2019).

The originality of the AGDI consists of its measuring gender equality through two components. The GSI measures the quantitative aspects of a country's gender regime. The AWPS qualitatively assesses and monitors government's progress on international and regional declarations, charters, conventions and protocols affecting women's rights, including the Beijing Platform for Action and CEDAW. The scorecard includes issues as harmful traditional practices, various dimensions of power, including power inherited from pre-colonial times, violence against women, land rights and conflict prevention. The scorecard uses a three-point scale, 0, 1, and 2.^38^

### 3.1. The Gender Status Index, GSI

The GSI draws on capabilities, opportunities and agency/empowerment to measure gender gaps in three key dimensions with additional sub dimensions: social power (health, education), political power (public sector, civic activities), and economic power (income, access to resources, time-use, and employment).

The social power block consists of two components: education (with sub-components for enrolment, dropout, literacy) and health (child health, HIV/AIDS prevalence, time spent out of work). The economic power block is divided between three components: income (with sub-components for wages and income, time-use or employment) and access to resources (means of production and management). The political power block has two components: public sector and civil society. After the pilot, the number of indicators was set at 44 (see Annex for the detailed list of indicators).

The lack of data for some indicators or sub-components in some countries was dealt with by comparing the index for those countries where the data were available, especially for time-use (at the time of the first pilot round, only three countries had carried out a time use survey: Benin, Madagascar, and South Africa).

Findings on the GSI and especially the economic power block and its components and sub-components show that the index and sub-indices are not dependent on the GDP. In the report of the pilot survey (UNECA, 2009), South Africa, Ghana, Tanzania and Uganda had GSIs in close range with their GDI, HDI, and GDP, but Madagascar, Mozambique and Burkina Faso were ranking much higher in the GSI. For instance, Madagascar ranked high in the GSI and low in the HDI. Gender parity was reached for many indicators but at a very low absolute level and with poorer performances in the economic and political power blocks: in such cases, the AWPS clearly demonstrated the severe lacks in many dimensions of the scorecard.

### 3.2. The African Women's Progress Scorecard, AWPS

The AWPS complements the GSI. Its first version had four blocks. Three blocks are similar to those of the GSI, social, economic, and political power. The fourth block is women's rights, focusing on CEDAW and the Women's Protocol of the African Charter of Human and People's Rights. The four blocks of the AWPS receive the same weight in the calculation of the AWPS; the indicators within each block receive the same weight in the block. The scorecard is a measure of government policy performance regarding women's advancement and empowerment. It includes issues such as harmful practices, violence against women, maternal mortality, contraception, HIV/AIDS, school dropouts, women's land rights and other economic and political issues. A country's performance on various ILO conventions and policies is scored, as is progress on the implementation of UN resolution 1325 on conflict resolution. These issues are listed on the vertical axis (32 indicators). On the horizontal axis 13 performance indicators are listed, from ratification, to budget, and monitoring (see table in Annex). A guidebook was prepared with scoring instructions. Countries are expected to produce extensive reports on these issues, justifying their scoring.

The evaluation after the 2004–2005 pilot phase revealed that rich country reports were produced but that scoring of the AWPS was a sensitive issue. Patriotic feelings intervened in some countries to produce an artificially high score. A comparison of the extensive country reports exposed the most glaring discrepancies. An intensive process of consultations between UNECA and the country teams followed in which such issues were discussed, yet inconsistencies remained. The UNECA team never imposed their own scoring on the country teams. Despite this divergence on scoring, the country teams all found the AWPS a valuable instrument that demonstrated the achievements and gaps in government policies. The narratives provided in the reports displayed very rich materials, which made comparisons between the countries possible. The lengthy reports made the AWPS transparent and a very useful tool for both NGOs and governments. The collaboration of experts from various disciplines in the country teams stimulated a better understanding on gender issues among all participants.

Another point raised was that prevalence of a particular issue (for instance HIV/AIDS) and scoring on policies related to that issue may not always run parallel. It was noted that countries with a low prevalence of HIV/AIDS might score low on government performance, indicating it was not a priority issue of the government. Scoring in the AWPS may be lower on an area that is not very disadvantageous for women, while it may be higher on an area in which women experience many problems. Thus, low AWPS scores do not always point to the need to increase government attention to a particular issue. Another bias was introduced when country teams counted contributions of foreign donors to particular issues (such as the school dropout for girls) instead of (or adding to) the government contribution. In such a case the AWPS score would show a positive bias.

The comprehensive and influential African Women's Report reporting on the pilot phase appeared in 2009. The gap between the first trials and the appearance of the report indicates the complexity of the process of data collection, scoring and analyzing the GSI and the AWPS.

A revised booklet on the AWPS was produced in 2010, which was used for the third and fourth groups of African countries that would implement the AGDI. This revision made the AWPS more comprehensive, but also more lengthy and costly to compute. On the horizontal axis two more indicators were introduced (capacity enhancement and accountability/transparency), while the vertical axis was increased from 32 indicators to 35. The major changes were including indicators on harmful practices (such as FGM, early marriage or widow inheritance), safe abortion and the participation of women in traditional governance. Other indicators were made up to date.^39^ Also added was the African Union's Solemn Declaration on Gender Equality in Africa. The indicators on violence against women were more sharply defined.

### 3.3. Use of national data

One of the specificities of the AGDI, especially the GSI, was to mainly rely on national data, rather than on international databases. The idea was to generate an internal demand for adequately-tailored indicators, sensitize producers and users at national level on the importance of gender statistics and show that mobilization at national level can prove effective and efficient for updating and providing the required indicators and variables. National Statistical Offices were duly involved and associated with the work of national AGDI committees. As noted above, the advantages of using national data are the following. First, national data take time to reach international databases; second, countries may not produce an indicator and the indicator is produced at international level, possibly leading to misinterpretations (see above the example on informal employment); third, the international database may decide to rectify national data without informing or interacting with the country.

### 3.4. Expansion of the AGDI

**Figure 1:**
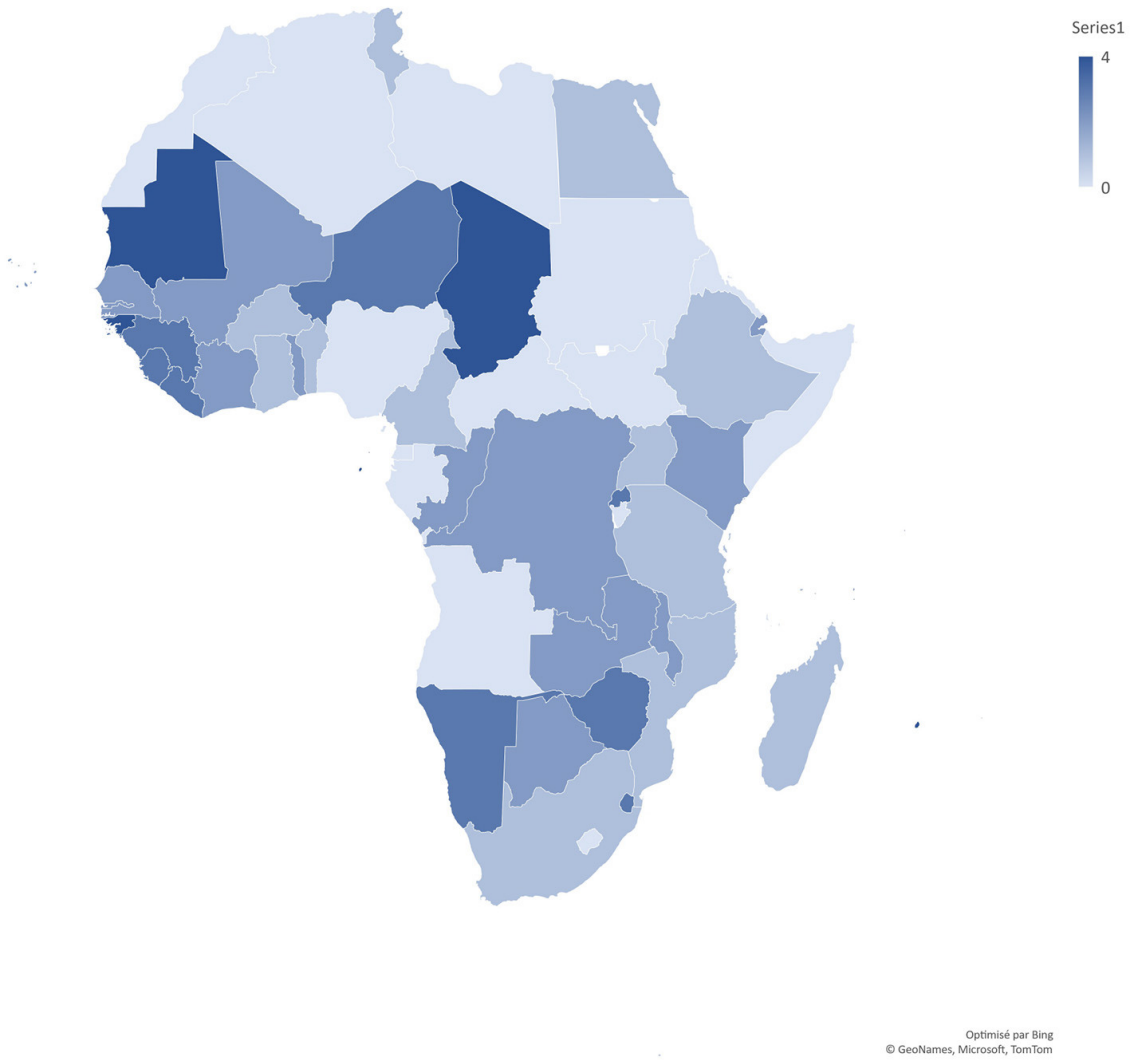
AGDI countries across implementation of the four rounds. Pilot (round 1, 2004–2005: 12 countries): Benin, Burkina Faso, Cameroon, Ethiopia, Egypt, Ghana, Madagascar, Mozambique, South Africa, Tanzania, Tunisia, and Uganda. 2nd round (2010: 13 countries): Botswana, Cabo Verde, Republic of Congo, Côte d'Ivoire, Djibouti, Democratic Republic of Congo, the Gambia, Kenya, Malawi, Mali, Senegal, Togo, and Zambia. 3rd round (2015: 10 countries): Eswatini, Guinea, Liberia, Namibia, the Niger, Rwanda, Seychelles, Sierra Leone, South Africa, and Zimbabwe. 4th round (2016: 5 countries): Chad, Guinea-Bissau, Mauritius, Mauritania, and Sao Tome and Principe.

The AGDI pilot study was conducted in 2004–2005 in 12 countries and the findings published in the African Women Report 2009 (UNECA, 2009), then extended in 13 more countries in 2010 with findings published in the AGDI II Regional Report (UNECA, 2017a). The two last rounds in 2015 and 2016 were extended to 10 (with one country repetition: South Africa) and five more countries respectively, with findings published in the AGDI III Regional report (UNECA, 2019) (Figure 1).^40^

### 3.5. Economic indicators

As mentioned, one of the main originalities of the AGDI process was to select some indicators that were neither readily nor widely available at the time when the study was conducted. This concerned the indicators on informal work, on unpaid care work and on the wage/income gender gap. Informality is an ambivalent concept: it is a means for women (and men) for achieving higher employment rates and at the same time a source that provides jobs of lower quality and low-paid. Therefore, the gender gap for this indicator cannot be interpreted without a look at the gender gap in unpaid care work (and total work) and in wage/income from employment.

The inclusion of these three indicators is part of the pro-active approach of AGDI and in this respect one can say that it paved the way for some of the SDGs' indicators that came later on. The AGDI early positioned itself in the strategy of the 5 Rs (Recognize-Reduce-Redistribute-Reward-Represent) designed to tackle inequalities in unpaid and paid care work.^41^

#### 3.5.1. Informal work

Today, data on informal employment disaggregated by sex are widely available, but this was not the case in the period 2000–2004, when the AGDI was designed and validated. This was long before the SDGs included the topic as one of its 231 indicators. It was however felt that the African labor markets could not be properly described through the usual labor force and employment indicators, especially as regards women whose presence in informal work was presumably strong. As a matter of fact, as was demonstrated later on, not only is Africa the region with the highest rate of informal employment, the share of women in informal employment is the highest in Africa and on this continent female informal employment rates are the highest (ILO, 2018). Given that labor force surveys remain scarce in Africa, the quest for the indicator on informal employment mobilized several types of household surveys, especially the living conditions surveys, the format of which may vary widely from country to country. Hence a huge heterogeneity of sources. Presently, informal employment data are available for 47 African countries (Charmes, 2019a).

#### 3.5.2. Time-use and unpaid care work

The AGDI is the first index explicitly seeking to overcome the underestimation and invisibility of women's paid and unpaid work (UNECA, 2011). It does so by including time-use data on market work; non-market economic activities (e.g., subsistence work) or as an unpaid family worker in market economic activities; and finally, unpaid domestic, care, and volunteer work. As such, it is the first gender equality index to measure the gendered division in unpaid reproductive labor, effectively strengthening its policy relevance.

**Figure 2:**
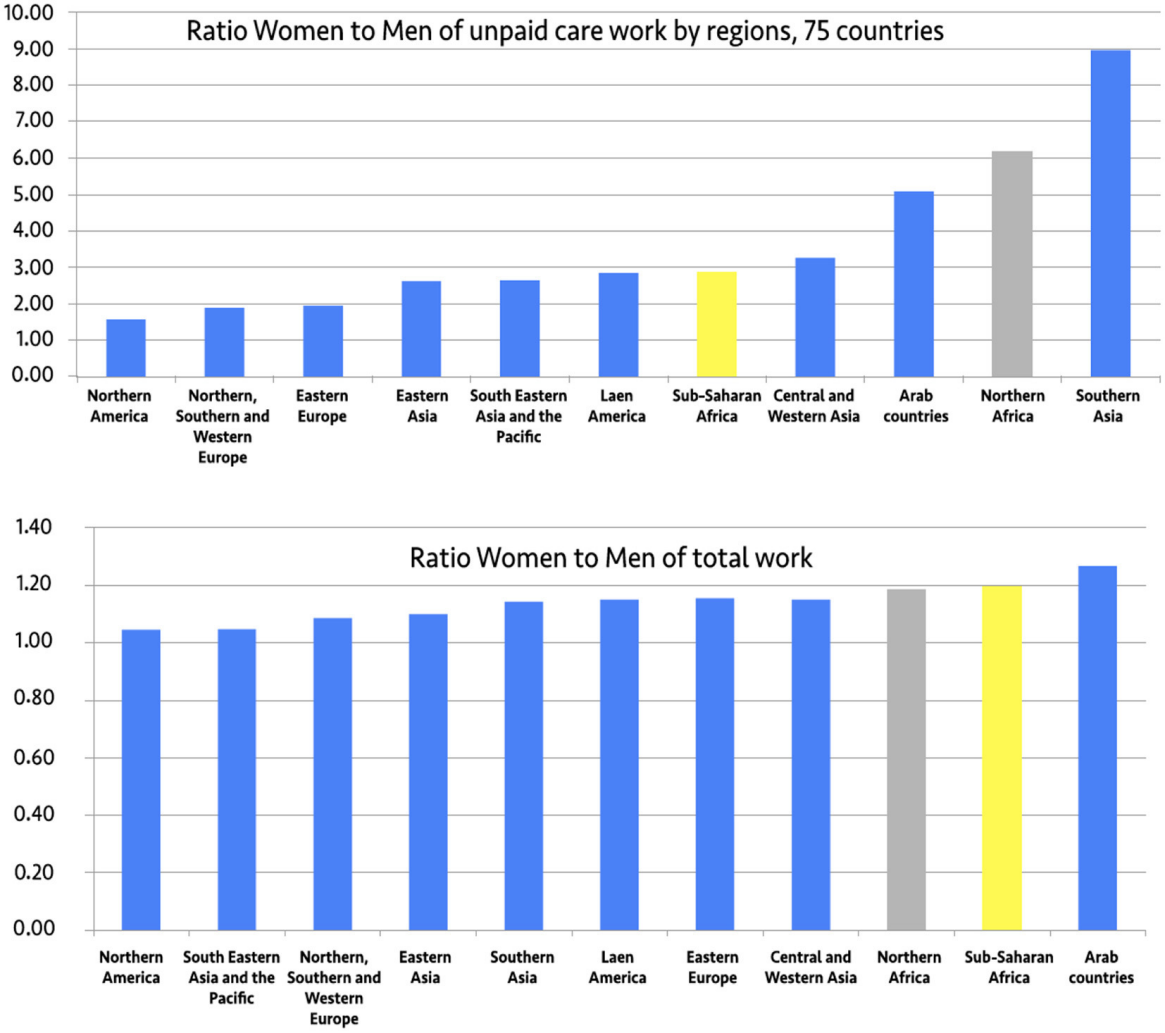
Gender gaps in unpaid work and total work by region. Source: Charmes (2019a,b).

Initially—and ideally—gender gaps in time spent in unpaid care work (household chores and caring for children and adult family members) and in paid work (formal and informal employment) were indicators deemed to enter into the computation of the AGDI. However, the small but increasing-number of African countries having implemented time-use surveys did not allow for keeping the indicator in the final calculation because it would have required too many imputations for missing countries. However, the national reports also mobilized data (of lower quality) from living conditions surveys that have more and more often collected data on time-use through short lists of unpaid activities. To date, 16 countries have implemented full time-use surveys (Algeria, Benin, Cape Verde, Cameroon, Ethiopia, Ghana, Kenya, Madagascar, Mali, Mauritius, Morocco, Senegal, South Africa, the United Republic of Tanzania, Tunisia, Uganda), and five of them have even repeated the exercise (Benin, Cameroon, Mauritius, South Africa, Tanzania). It is interesting to highlight the findings for the African countries and compare them with other regions in the world. As shown in the two charts below, the burden of women's unpaid care work equals 2.86 times the burden for men in sub-Saharan Africa, and up to 6.18 times in Northern Africa. Including paid work, the burden of women's total work represents 1.2 times the total of men's work in sub-Saharan Africa and 1.19 times in Northern Africa, positioning African women among those who suffer the heaviest burden of work in all regions (Figure 2).

#### 3.5.3. Wage/income gender gap

In recent years all across the continent, data on wages and income from work have been more and more engendered. The number of household surveys and informal enterprises surveys capturing such indicators has increased and administrative statistics on civil servants or employees (national social security funds) as well as formal enterprises' surveys have more often disaggregated their data by sex. Among the 54 African countries in 2019, 35 have been able to provide or make available in national publications such data, which allow for the calculation of gender gaps. However, data on this indicator are still far from being homogeneous: in some countries the indicator is covering civil servants only, or all employees of the public sector, or all employees of the whole formal sector (including public and private). In other countries, data disaggregated by sex are only available for the informal sector (both for wages and entrepreneurs' income), and for a few countries they are available for each subsector and for the whole economy. Sources are also very diverse: including administrative records such as civil service payrolls, social security registers, enterprise surveys (on the formal sector or on the informal sector), and household surveys especially living conditions surveys (but the earnings of household heads have not been taken into account as they are a different indicator): today the wage gender gap can be calculated for 35 countries from this unique source. Despite this heterogeneity, it is important to stress the efforts made and the progress accomplished that allow the demonstration of a gender gap of 26.0% (female earnings representing only 74.0% of males' earnings), with considerable variations among countries. Such a result demonstrates that the 0.75 coefficient applied by the UNDP's HDI and by the WEF for imputation to missing countries seems to be adequate, though calculated on a small number of mainly developed countries.

### 3.6. How the issue of ranking was overcome

As already mentioned, countries fear rankings and do not always welcome the results of such an exercise, so much prized by international institutions. To deal with this prevention, AGDI never put the focus on the overall ranking, but it rather highlighted dimensional rankings or at indicator level, emphasizing as far and as often as possible specific trends. The AUC—which is a more political institution—went even further, by nominating a set of countries in each dimension of the scorecard and giving awards to the best performing, based on the scorecard.

### 3.7. The way forward

The latest revision of the AGDI and the AWPS took place in 2019. By that time 39 countries had gone through the process, producing rich reports. In 2019 the third Regional Synthesis Report of the AGDI had been published, entitled Measuring Gender Equality and Women's Empowerment in Africa (UNECA, 2019). In an evaluation session it was concluded that the AWPS needed to be streamlined. It had to become even better suitable to fulfill a country's monitoring obligations under international and regional commitments. It had to focus more on the SDGs which had been adopted in 2015, in relation to the UN 2030 Agenda for Sustainable Development. The African Agenda 2063 also had to be incorporated. After lengthy discussions it was decided that scoring should continue to consist of the three-point scale but that criteria needed to be more sharply defined. To ensure greater consistency across countries it was recommended that one or more of the experts in the national team may be regionally based.

The horizontal indicators were reduced to four main performance indicators, ratification/law, policy/plan, institutional mechanism/coordination and implementation. The redefined AGDI was articulated around seven dimensions of a right-based approach (with 40 indicators for the GSI and 21 for the AWPS): (1) Rights to non-discrimination and equality, includes right to marry and rights in marriage; (2) Right to live free from gender-based violence against women; (3) Right to education; (4) Right to health; (5) Right to work and rights at work; (6) Right to an adequate standard of living, including right to food and right to social security; and (7) Right to participate in political and public life. The Annex contains the 2020 GSI and AWPS. Some changes in the implementation of the process of data collection and analysis at country and regional level were introduced. This included the use of quality panels to review the national reports. These panels would consist of staff from ECA, and African and international experts. In order to bring a sub-regional perspective to the review, staff of ECA's sub-regional offices would be included on the panels, as well as one expert from each sub-region. This new approach was tested in Namibia and the Seychelles.

In light of the development of new indices discussed above, UNECA decided to take stock of the AGDI and to ensure that this index—which by many aspects was a precursor, if not an inspirational tool (though it is rarely referred to in the literature)—remains relevant and keeps its place besides more classical indices focussing on ranking because of its uniqueness: combining the GSI and the AWPS, relying on national data and ensuring ownership of the procedure by national advisory panels. To this aim a mixed group of experts and official national representatives was convened in the fall 2022 at UNECA headquarters to decide on the next steps.

The revised structure of the GSI and the AWPS was discussed, and the experiences of Namibia and the Seychelles assessed. The new structure is in better alignment with the SDGs but this does not mean that the AGDI will less rely on national data, a characteristic that remains central for the exercise, as well as the ownership of the procedure by national advisory panels whose composition and roles should be redefined: their better institutionalization within national statistical and reporting frameworks, their sustainability and empowerment, for a better dissemination and regular updating. In other words, it would be necessary to make sure that the persons responsible for reporting to international protocols are members of the advisory panels, together with representatives from ministries providing data, statistical experts and representatives from Civil Society Organizations.

In the discussions it was proposed that the AGDI takes the form of a dashboard rather than a composite index. It was also felt that in view of the serious consequences of climate change a block on that topic be added. It was also felt that the four performance indicators of the AWPS were perhaps too few, as important aspects of government performance, such as accountability and the provision of a sufficient budget now could not be assessed.

## 4. Conclusion

The three regional reports produced by the ECA and the many country reports generated by the country teams demonstrate that the AGDI is a useful tool in producing engendered data for policy making, reporting on a country's international commitments on gender issues and in knowledge production. The AGDI is built on a solid theoretical framework incorporating women's empowerment and statistical analysis on gender relations which indicates that theoretical adequacy need not be compromised by concerns on data availability. African gender experts have been heavily involved in its design and have been responsible for its implementation. Their involvement has reduced the dependence on biases from the global North which so often influence indices. Too often data availability guides the design of indices. The empty spaces in both the GSI and the AWPS serve a purpose: stimulate national statistical bureaus to start collecting those data. Most gaps were found in such indispensable indicators as time-use, access to resources, and political power. The AGDI has emerged as an effective advocacy tool for better data collection and to-date, there is an increasing number of African countries planning a national time-use survey.

The GSI is more independent of the GDP than measures such as the GDI and allows for an insightful comparison between African countries. The combination of quantitative and qualitative data allows for analysis of the effects of government policies on a country's unequal gender regime. The incorporation of African gender documents and indicators that are specifically relevant for African countries adds to its overall usefulness for the region. The AWPS qualitatively assesses country progress in ratifying and implementing (inter)national and regional conventions and declarations; in particular progress on the Beijing Platform for Action and the African Charter of Human and People's Rights (in the first two regional reports) helps African States to fulfill their international reporting obligations. The latest version of the AWPS reduces the considerable efforts spent on scoring, while keeping its overall utility, though some members of the 2022 advisory meeting deplored missing out on some information.

The GSI insists on the importance of unpaid reproductive labor, and its central role in gender inequality. Likewise, both the GSI and the AWPS cover a larger set of indicators in relation to power than other international indices. As the unequal power relations between the sexes is one of the most important sources of gender inequality, the relevance of these indicators is clear. The same goes for the indicators on gender-based violence. These are found in the AWPS, so that the commitment of governments to eradicate such violence is measured. As data on incidence are notoriously unreliable this way of emphasizing the topic was found to be effective. Though the use of national data instead of international data sets was found to have various advantages, some problems also came to the fore, when different concepts and definitions were used. The deployment of regional advisors to the country teams reduced the tendency of some state to score their country's performance optimistically, this remains an area of concern.

Since the launch of the first two gender indices, the GDI and the GEM, in 1995, great progress has been made to improve gender statistics. The AGDI is one of the most promising indices to date. Yet several topics require continued attention. This includes collecting more reliable and relevant data on gender-based violence and political power. Data collection on informal labor has greatly improved in recent years [with indicators for 33 African countries in the ILO database (ILO, 2018) and for 47 countries in Charmes (2019a)]. However, the most recent year is rather old for some of them. As to time-use data, we mentioned that 16 African countries have conducted time-use surveys, four of them repeated them and data collection is planned for five more. An acceleration of data collection on time use and unpaid work can be observed in the recent period with the support of UN Women that makes recognition of unpaid work a priority of its conceptual and strategic 3R (Recognition-Reduction-Redistribution) framework,^42^ especially in Africa. There is no doubt that the AGDI played an important role in the prioritization of time-use in data collection by National Statistical Offices.

Since the first country reports based on the AGDI appeared, the design and the implementation of the AGDI have been considerably improved. The AGDI provides most data needed for reporting on CEDAW and the SDGs and for national policy making on gender issues. The wealth of data generated also offers scope for comparative analyses, between the GSI and the AWPS, between regions and countries, and over time (as exemplified by South Africa that produced two reports). The AGDI can also be implemented at a subnational level, which increases its relevance in large, complex countries such as Ethiopia or Nigeria.

The AGDI inspired the two African indices that were designed in the mid-2010s (the AGI of AfDB and the AGS of AUC, discussed above) and reflections were conducted toward revision, harmonization and coordination of the approach by both institutions with UNECA. A tentative revision of the AGDI was proposed in 2020, aligning the indicators of the GSI and AWPS with the human rights approach and adopting some of the SDG's indicators (see Annex).

Despite pressures toward giving up its compilation and leaving the place to the new AfDB-UNECA AGI, the members of the 2022 advisory panel felt that the AGDI should continue because the feedback from partner countries is extremely positive; they appreciate the ownership of the process that strengthens their ability to report to their international commitments and they engage in the process on a voluntary basis. At national level, this nationally-owned tool is preferred to the recourse to international instruments.

Ranking countries remains an interesting and tricky business, boosting or deflating national egos. However, it is an indispensable process for international reporting, for statisticians and for researchers. But for civil society and civil servants who want to lift people out of poverty and fight for equality and human and women's rights, much more detailed, national-level and transparent data are important. The combination of qualitative and quantitative data on social, economic and political issues, the inclusion of rights-based information and government performance, the use of national data sources and the transparent way of calculating, have made the AGDI an inspiring and useful tool. It will continue to be improved as geopolitical events evolve and new information is needed.

## Author contributions

All authors listed have made a substantial, direct, and intellectual contribution to the work and approved it for publication.

## Footnotes

^1^See Sen (1985, 2001).

^2^It has become the geometric mean since 2010 in order to limit compensation between dimensions.

^3^To date it has been ratified by 189 states, with over 50 states having declared a reservation. The Optional Protocol, which allows complaints from individuals to be heard, is signed by 80 countries.

^4^Among the 231 unique SDG's indicators, some 53 are gender indicators; if indicators relating to population—that can be disaggregated by sex—are included the number goes up to 80 (UN Statistics Division, 2019; UN Women, 2022).

^5^This project was preceded by a Technical Workshop on Building a Framework for Measuring Gender Equity in Bangalore in May 1996. Wieringa (1996) argued that discussions on human agency, sexual politics and cultural and religious practices were missing.

^6^See also Bardhan and Klasen (1999). Klasen (2006) elaborated this critique and proposed some solutions.

^7^Dijkstra and Hanmer (2000) produced a scatterplot of the GDI and the GDP per capita which demonstrates that there are very few outlier countries. Eight dimensions were proposed for the GEI: gender identity; autonomy of the body; autonomy within the household; political power; social resources; material resources; employment and income and lastly time use. The WEM distinguished six spheres (physical, sociocultural, religious, political, legal and economic) and six levels (personal, household, community, state, region, and global).

^8^These are economic participation and decision-making, political participation and decision-making, and power over economic resources.

^9^See also Beteta (2006) for a further discussion on women's empowerment.

^10^An added problem of the income indicator is that for many countries a proxy was used of female wages being 75% of male wages.

^11^See also Schmid (2021), who analyses 17 gender indices. This number does not include the subnational gender index developed for Indonesia by the women's network APIK, the APIK Gender Justice Index (AGJI) which is designed after the methodology introduced by the African AGDI (Wieringa, 2019).

^12^ILO: ILOSTAT (https://ilostat.ilo.org/).

^13^UNESCO Institute of Statistics (https://uis.unesco.org/).

^14^WHO Global Health Observatory (https://www.who.int/data/gho).

^15^World Bank: World Development Indicators (https://databank.worldbank.org/source/world-development-indicators).

^16^UN SDGs (https://unstats.un.org/sdgs/dataportal).

^17^In this African country, there is no official definition of informal employment and none estimate is generated at national level. Given the availability of micro data, the international database generated its own estimate of informal employment for the country (53%), an estimate that found its way to the SDGs database, though not provided by the country. An in-depth review of the national data by one of the authors of this article revealed the overestimate of the indicator included in the SDGs database (the indicator was 33%).

^18^The Index is computed as follows. First, achievement-adjusted gender gaps are computed for all constituent metrics. Next, these achievement-adjusted gaps for closely related metrics are combined to obtain sub-domain scores. Then, subdomain scores are combined to obtain domain scores. Finally, the domain scores are combined to obtain the overall Gender Equality Index score.

^19^ https://eige.europa.eu/gender-equality-index/2013

^20^ https://eige.europa.eu/publications/gender-equality-index-2015-measuring-gender-equality-european-union-2005-2012-main-findings#downloads-wrapper

^21^See also Mohamad and Wieringa (2013) and Wieringa (2019).

^22^Only in 2022 political power was mentioned in the Annex to the GEI.

^23^Hausmann et al. (2006), p. 4.

^24^See for a discussion of the use of a wider set of indicators on power Wieringa (2019). See also Beneria and Permanyer (2010).

^25^On these aspects, see also the World Bank publication *Women, Business and the Law* and its time-series database (World Bank, 2022).

^26^World Economic Forum (2021).

^27^We can recall here that this 0.75% comes from the HDI and that it is the wage gap computed on some 55 countries (of which 23 developed) at the time of the 1995 Human Development Report.

^28^ https://hdr.undp.org/data-center/thematic-composite-indices/gender-inequality-index#/indicies/GII

^29^Zaluchu (2018).

^30^See for instance Branisa et al. (2013).

^31^See Cohen (2015) for a discussion on qualitative vs. quantitative sociological methods.

^32^The initiative was taken by Josephine Ouédraogo at the time Director of the UNECA gender division (African Center for Gender and Development, ACGD). It was fully supported by K.Y. Amoako, Executive Secretary of the ECA. In 2005 Ouédraogo became Deputy Executive Secretary of the UNECA. Since then Thokozile Ruzvidzo assumed the position of Director of the ACGD; she was succeeded by Gonzaque Rosalie in the supervision and monitoring of AGDI. From the start Jacques Charmes and Saskia Wieringa were involved as consultants in the design of the index.

^33^Sen (1989).

^34^See for instance Rubin (1975). See also Butler (1993).

^35^Duflo (2012).

^36^Scott (1989).

^37^See Simandan (2019a,b) for a further theorization of the concept of situated knowledge.

^38^Governments' policy performance is scored with a three-point scale: 0 for no progress: 1 for moderate progress; and 2 for sufficient progress. Scoring is done in country teams, which should include members of both government agencies and of the civil sector, and consists of both gender specialists and economic and statistical experts.

^39^Such as the UN Security Council Resolution 1325 on Women, Peace and Security, which in the meantime had been joined by Resolutions 1820, 1888, and 1889.

^40^An updated technical report contained the most recent definitions and directions for scoring (UNECA, 2017b).

^41^Designed by Elson (2000) for UNIFEM Progress of the World's Women 2000, the 3Rs strategy (Recognize-Reduce-Redistribute unpaid care work) was later completed by two more Rs.

^42^And 5 R if the care sector is taken into account globally with Reward and Representation of the paid care workers (Addati et al., 2018).
