# Supplementary material for: The African Gender and Development Index: an engendered and culturally sensitive statistical tool
Source: Front Sociol. 2023 Jun 1;8:1114095. doi: 10.3389/fsoc.2023.1114095 (PMC10267327; doi:10.3389/fsoc.2023.1114095)
Supplement: Supplementary file 1 [file Table_1.pdf]

The Gender Status Index (GSI) of AGDI (2010 version)

Block	Component	Sub-component	INDICATOR		Female	Male
Social power **'Capabilities'**	Education	Enrolment	Early childhood enrolment			
			Primary enrolment rate (net)			
			Secondary enrolment rate (net)			
			Tertiary enrolment rate (gross)			
		Completion	Proportion of pupils starting grade 1 who reach last grade of primary			
		Literacy	Literacy rate of 15-24 years old			
	**Health**	Child health	Stunting under 5 using minus 2 standard deviation			
			Underweight under 5 using minus 2 standard deviation			
			Mortality under 5			
		HIV/AIDS	HIV/AIDs prevalence among 15-24 years old			
			Access to anti-retroviral treatment			
**Economic power 'Opportuni-ties'**	Income	Wages	Wages in agriculture			
			Wages in civil service			
			Wages in formal sector (public and/or private)			
			Wages in informal sector			
		Income	Income from informal enterprise			
			Income from small agricultural household enterprise			
			Share of women under the poverty line			
	**Time-use and employment**	Time-use	Time spent in market economic activities (as paid employee, own-account or employer)			
			Time spent in non-market economic activities or as unpaid family worker in market economic activities			
			Time spent in domestic, care and volunteer activities			
		Employment	Share of women in non-agricultural wage employment			
			Youth unemployment rate			
	**Access to resources**	Means of production	Ownership of	rural land/farms		
				urban plots/houses		
				livestock		
			Access to credit (commercial and micro-credit)			
		Management	Employers			
			Own-account workers			
			High civil servants (class A)			
			Members of professional syndicates			
**Political power 'Agency'**	**Public sector**		Members of parliament			
			Cabinet ministers **			
			Higher positions in civil service and parastatals			
			Employment in the security forces			
			Judges of	higher courts		
				lower courts		
				traditional and religious courts		
			Members of local councils			
			Number of male/female traditional rulers			
	**Civil society**		Senior positions in	Political parties		
				Trade unions		
				Employers' associations		
				Heads or managers of NGOs		

The structure of the revised Gender Status Index 2020 version

Indicator	Female	Male	Gender Status Index
**A. Rights to non-discrimination and equality, includes right to marry and rights in marriage (1 indicator)**			
1. Proportion of population reporting having personally felt discriminated against or harassed in the previous 12 months on the basis of sex			
**B. Right to live free from gender-based violence against women (1 indicator)**			
2. Proportion of young women and men aged between 18 and 29 years who experienced sexual violence by age 18			
**C. Right to education (6 indicators)**			
3. Early childhood enrolment			
4. Primary enrolment rate (net)			
5. Secondary enrolment rate (net)			
6. Tertiary enrolment rate (gross)			
7. Proportion of pupils starting primary grade 1 who reach last grade			
8. Literacy rate of 15-24 year olds			
**D. Right to health (3 indicators)**			
9. Under-five mortality rate			
10. Number of HIV infections per 1,000 uninfected population			
11. Access to anti-retroviral treatment			
**E. Right to work and rights at work (6 indicators)**			
12. Proportion of time spent on unpaid domestic and care work			
13. Share of the population in non-agricultural wage employment			
14. Proportion of informal employment in non-agriculture employment			
15. Average hourly earnings			
16. Proportion of employers			
17. Unemployment rate			
18. Proportion of youth (aged 15-24 years) not in education, employment or training (NEET)			
**F. Right to an adequate standard of living, including right to food and right to social security (8 indicators)**			
19. Proportion of population living below the national poverty line			
20. Proportion of population covered by social protection floors/systems			
21. Proportion of total adult population with secure tenure rights to land, with legally recognized documentation and who perceive their rights to land as secure			
22. Ownership of livestock			
23. Access to credit			
24. Average income of small-scale food producers			
25. Prevalence of stunting (height for age among children under 5 years of age			
26. Prevalence of wasting (weight for height) among children under 5 years of age			
**G. Right to participate in political and public life (15 indicators)**			
27. Cabinet ministers			
28. Members of parliament			
29. Higher positions in civil service and parastatals			
30. Employment in the security forces			
31. Judges of higher courts			
32. Judges of lower courts			

33. Judges of traditional and religious courts			
34. Members of local councils			
35. Traditional rulers			
36. Senior positions in political parties			
37. Senior positions in trade unions			
38. Senior positions in employers' associations			
39. Senior positions in non-governmental organizations			
40. Members of professional syndicates			

AWPS 2004 Version

			Ratification	Reporting	Law	Policy	Development of a plan	Targets	Institutional mechanism	Budget	Human resources	Research	Involvement of civil society	Information & dissemination	Monitoring & evaluation	Total	%
**WOMEN' S RIGHTS**	CEDAW	Ratification without reservation															
		Optional protocol					x	x	x	x	x	x	x	x	x		
		Art 2															
		Art 16															
	African Charter of Human and People's Rights – Women's Rights protocol – harmful practices			x													
**SOCIAL**	Beijing Platform of Action		x														
	Violence against Women	Domestic violence	x														
		Rape	x														
		Sexual harassment	x														
		Traffic in women															
	African Charter on the Rights of the Child art XXVII																
	Health-ICPD POA Plus Five	STI's	x	x													
		HIV/AIDS	x	x													
		Maternal mortality	x	x													
		Contraception	x	x													
	2001 Abuja Declaration on HIV/AIDS and women		x	x													
	Education	Policy on girl school dropouts	x	x													
		Education on human/women's rights	x	x	x												

**ECONOMIC**	ILO	Convention 100															
		Convention 111															
		Convention 183															
		Policy on HIV/AIDS	x	x													
		Engendering NPRS	x	x	x												
		Access to agricultural extension services	x	x	x												
		Access to technology	x	x	x												
	Equal access to land		x	x													
**POLITICAL**	UN 1325 conflict resolution																
	Beijing PFA effective and accessible national machinery		x		x												
	Policies	Support for women's quota and affirmative action	x	x	x												
		Decision making positions within parliament/ministries	x	x													
		Gender mainstreaming in all departments	x	x	x												
Total score			x	x	x	x	x	x	x	x	x	x	x	x	x		

AWPS 2020 version

	Ratification/law	Policy/plan	Institutional mechanism/	Implementation	Total
**A. Rights to non-discrimination and equality, includes right to marry and rights in marriage (4 indicators)**					
1. Gender equality provision in the constitution (Article 2 of CEDAW)					
2. No impediment to gender equality due to religious, traditional or customary regulations					
3. Gender equality in family code (Article 16 (1) of CEDAW)					
4. Optional Protocol to CEDAW					
**B. Right to live free from gender-based violence against women and girls (5 indicators)**					
5. Law on gender-based violence against women					
6. Marital rape					
7. Sexual harassment					
8. Prohibition of child marriage (Article 16 (2) of CEDAW					
9. Prohibition of female genital mutilation					
**C. Right to education (1 indicator)**					
10. School dropouts					
**D. Right to health (3 indicators)**					
11. Maternal mortality					
12. Unmet contraceptive needs					
13. Safe abortion					
**E. Right to work and rights at work (3 indicators)**					
14. Equal wages (ILO Convention on Equal Remuneration, 1951 (C100))					
15. Parental leave					
16. Childcare					
**F. Right to an adequate standard of living, including right to food and right to social security (2 indicators)**					
17. Support for women’s ownership					
18. Extension services/training					
**G. Right to participate in political and public life (3 indicators)**					
19. Gender issues mainstreamed in policies on climate change					
20. National machinery					
21. Support for women’s quotas and affirmative action					
